# Identification and Characterization of Seven Glutathione *S*-Transferase Genes from Citrus Red Mite, *Panonychus citri* (McGregor)

**DOI:** 10.3390/ijms141224255

**Published:** 2013-12-13

**Authors:** Chong-Yu Liao, Kun Zhang, Jin-Zhi Niu, Tian-Bo Ding, Rui Zhong, Wen-Kai Xia, Wei Dou, Jin-Jun Wang

**Affiliations:** Key Laboratory of Entomology and Pest Control Engineering, College of Plant Protection, Southwest University, Chongqing 400715, China; E-Mails: leochongyu@gmail.com (C.-Y.L.); zhangkunbest@gmail.com (K.Z.); jinzhiniu@yahoo.com (J.-Z.N.); tianboding@gmail.com (T.-B.D.); zhongrui19890326@gmail.com (R.Z.); wenkaixia0409@gmail.com (W.-K.X.); anwdou@gmail.com (W.D.)

**Keywords:** *Panonychus citri*, glutathione *S*-transferase, gene expression, pyridaben, fenpropathrin

## Abstract

The citrus red mite, *Panonychus citri* (McGregor), is a global citrus pest, and has developed severe resistance to several types of acaricides. However, the molecular mechanisms of resistance in this mite remain unknown. In this study, seven full-length cDNAs encoding glutathione *S*-transferases (GSTs) genes were identified and characterized in *P. citri*. The effects of pyridaben and fenpropathrin exposure on the expression of these genes were also investigated. Phylogenetic analysis revealed that the seven GSTs genes in *P. citri* cloned in this study belong to three different cytosolic classes, including four in mu, two in delta and one in zeta. Among these seven GSTs genes, the relative expression level of *PcGSTm1* was significantly higher in adult than in the other life stages (egg, larvae and nymph). Compared with the control, the mRNA levels of the seven GST genes did not change significantly following exposure to pyridaben at LC_10_. However, RT-qPCR results showed that, when exposed to LC_10_ of fenpropathrin, six GSTs gene (*PcGSTm1*, *PcGSTm3*, *PcGSTm4*, *PcGSTd1*, *PcGSTd2* and *PcGSTz1*) transcripts increased in a time-dependent manner. This is the first insight into the molecular characteristics of GSTs gene cDNAs in *P. citri*. The elevated GSTs gene transcripts following exposure to fenpropathrin might be one of the mechanisms involved in detoxification of this acaricide.

## 1. Introduction

As one of the three major detoxification enzymes, glutathione *S*-transferases (GSTs; EC 2.5.1.18) are phase II metabolic enzymes capable of conjugating reduced glutathione to electrophilic centers of a wide range of exogenous and endogenous compounds [1]. GSTs can metabolize insecticides to more readily excreted water-soluble compounds by facilitating reductive dehydrochlorination or through conjugating with reduced glutathione [2]. It has been reported that GSTs play important roles in the development of pesticide resistance, disease pathogenesis, and cellular stress responses in mites and ticks [3–5]. For example, increases in GST transcripts have been investigated in scabies mites exposed to ivermectin [6]. Elevated levels of GSTs might play a protective role in *Nilaparvata lugens*, an insect species, following pyrethroid exposure [7]. GSTs can be induced not only by general inductive agent of this enzyme, but also by various insecticides [8,9]. In addition, GST gene expression levels have been shown to decrease in locusts when exposed to different concentrations of deltamethrin [10]. Thirty-five GST genes belonging to five different canonical GST classes (delta, epsilon, mu, omega and zeta) have been identified in the tick species *Ixodes scapularis*, which is a disease vector of veterinary and public health importance [11]. Annotation of the *Tetranychus urticae* genome has revealed the full extent of GSTs in Acari. The results of genome analysis showed that the delta and mu classes of GSTs exist as large gene clusters in two spotted spider mite GST superfamilies, but no epsilon class of GSTs has been identified in this species [12]. Moreover, 24 Unigene transcripts belonging to seven classes (delta, kappa, mu, omega, sigma, theta and zeta) have been identified from the transcriptome data of *Panonychus citri* [13]. Comparatively, in insects, GST superfamily genes have been clearly classified into at least six classes, including delta, epsilon, omega, sigma, theta and zeta [14].

The citrus red mite, *Panonychus citri* (McGregor) (Acari: Tetranychidae), is a major citrus pest distributed worldwide [15]. In the citrus orchards of southern China and Japan, the population has two peak time every year, one in early summer (June–July) and the other in autumn (October–November), while maintains low density during late summer and winter [16,17]. Moreover, owing to its unique biology and ecology, including a short life cycle, abundant progeny and arrhenotokous reproduction, the citrus red mite can survive frequent large-scale spraying of various acaricides/insecticides [18]. Since the first case of acaricide/insecticide resistance was reported in China in 1979, the citrus red mite has developed different degrees of resistance to various chemical classes of acaricides/insecticides (*i.e.*, pyrethroids, abamectin, bifenazate, mitochondrial electron transport inhibitor acaricides and spirodiclofen) [19–21].

Enhanced enzymatic detoxification and target-site insensitivity are major causes of resistance in Acari [18]. Additionally, cytochrome P450 monooxygenases (P450s), carboxylesterase (CarE) and GSTs are the major detoxification enzymes involved in the metabolism of pesticides before they reach the target site [1]. However, there is limited research into acaricide/insecticide resistance mechanisms in *P. citri* relative to that in the model organism of Acari, *T. urticae* [18]. There have been some recent studies on detoxification enzymes that might be involved in development of resistance in *P. citri*, such as two cytochrome P450 genes, *CYP4CF1* and *CYP4CL2* [22]. Similarly, the results of acaricide challenge experiments showed that CarE genes, *PCE1* and *PCE2*, may participate in the detoxification of avermectin and pyridaben [23]. By comparing the correlation coefficient of susceptibility of citrus red mite to pyridaben in nine field populations, along with their GST activities, it was suggested that GSTs could possibly be involved in detoxification of pyridaben in several field populations [24]. However, there is no available information about the relationship between GSTs and acaricides/insecticides at a molecular level in *P. citri*.

In our previous study, we identified 24 unique GST transcripts from annotation of the *P. citri* transcriptome [13]. To further characterize the molecular responses of GST genes to acaricides/insecticides in *P. citri*, in the current study we further obtained seven full-length GST cDNAs and carried out a detailed analysis of these genes. We then investigated potential differences in transcript expression levels that might be associated with acaricide metabolism and induction, and thus predicted functions of GST genes in *P. citri* by comparison with known GSTs in other arthropods.

## 2. Results and Discussion

### 2.1. Identification and Classification of *P. Citri* GSTs

**Figure 1.**
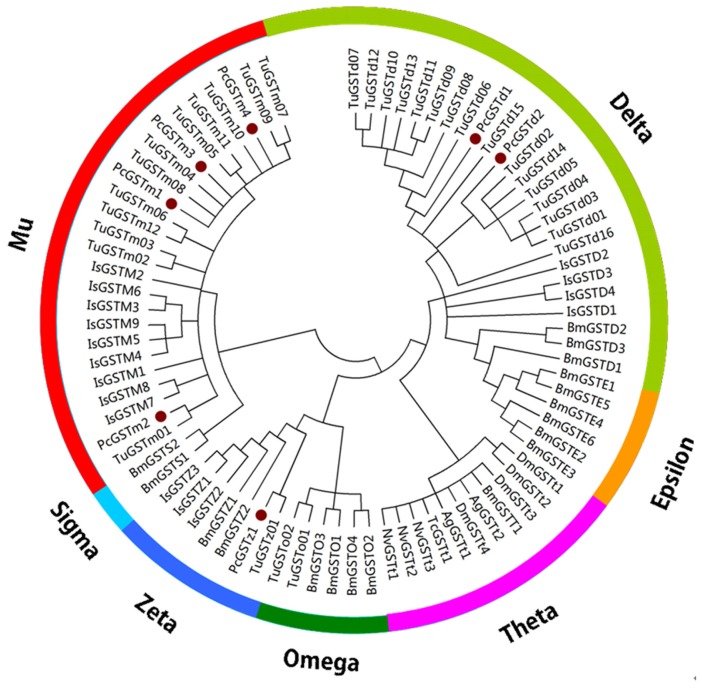
Phylogenetic relationship of 70 glutathione *S*-transferase (GST) proteins from six arthropod species, *Tetranychus urticae* (Tu, 31), *Ixodes scapularis* (Is, 16), *Bombyx mori* (Bm, 17), *Drosophila melanogaster* (Dm, 4), *Anopheles gambiae* (Ag, 1), *Nasonia vitripennis* (Nv, 3) and *Tribolium castaneum* (Tc, 1). Amino acid sequences were completely aligned using ClustalX, and a distance neighbor-joining tree was generated using MEGA 4.0. The seven *P. citri* GST genes are marked with filled brown circles.

In total, seven different GST transcripts were identified from the transcriptome database and were further aligned with the nucleotide sequences from genome datasets of *T. urticae* [25]. The complete, full-length cDNAs containing open reading frames were amplified by RACE-PCR, and the cDNA and deduced amino acid sequences were deposited in GenBank under the following accession numbers: JQ069034 (*PcGSTm1*), JQ069035 (*PcGSTm2*), JX846609 (*PcGSTm3*), JX846610 (*PcGSTm4*), JQ069033 (*PcGSTd1*), JQ069037 (*PcGSTd2*) and JQ069036 (*PcGSTz1*). Identity analysis and protein BLAST analysis of the predicted amino acid sequences of these GSTs suggested that the seven genes belonged to three different cytosolic classes, including four in mu (*PcGSTm1*, *PcGSTm2*, *PcGSTm3* and *PcGSTm4*), two in delta (*PcGSTd1* and *PcGSTd2*) and one in zeta (*PcGSTz1*) (Figure 1). The lengths of the deduced amino acid sequences of these seven *P. citri* GSTs varied from 217 to 224 a.a., and the predicted protein molecular weight ranged from 23.9 to 26.6 kDa, with theoretical isoelectric points of 5.18–7.65 (Table 1). The deduced amino acid sequence similarities were 11.4%–60.0% among all seven GSTs, 33.0%–60.0% among the four mu-class GSTs, and 53.2% between the two delta-class GSTs (Table 2). Among the different classes, the similarities of the deduced amino acid sequences were 11.4%–24.6% (Table 2). These results were consistent with the distribution of the seven putative amino acid sequences on the phylogenetic tree (Figure 1). However, based on transcriptome datasets, the remaining classes (kappa, omega, sigma and theta) of GST Unigene transcripts could not be amplified to full length in this study. This may be a result of low expression or the difficulty of designing primers according the restricted sequence lengths. However, the identified Unigene transcripts provide important information for a comprehensive understanding of the GSTs of *P. citri*.

### 2.2. Comparison of GSTs from *P. Citri* and Other Species

To date, all available GSTs have been classified into 13 different classes according to their sequence homology and molecular characteristics, and some GSTs are species-specific [26,27]. In Acari, total 35 GST genes belonging to seven classes (delta, epsilon, mu, omega, zeta, kappa and microsomal) have been found, based on analysis of the *I. scapularis* genome [11]. In addition, the genome dataset from *T. urticae* showed that all of the 31 cytosolic GST genes could be placed into four different classes: delta (16), mu (12), omega (2) and zeta (1) [12]. In this study, we only obtained seven full-length cDNAs encoding *P. citri* GST genes based on the transcriptome datasets, indicating that further investigation of remaining GSTs in *P. citri* is needed.

The delta class of GSTs is one of the two insect-specific GST classes, and members of this class can be found in the genomes of most insects, including *Drosophila melanogaster* [28], *Anopheles gambiae* [29], *Aedes aegypti* [30] and *Bombyx mori* [31] (Table 3). Although only two delta-class GSTs were obtained in the current study, the results are consistent with the previous prediction that this class of GSTs is likely to be widespread in Acari, as well as in *Sarcoptes scabiei* [32]. In addition, the popular understanding that delta-class GSTs are insect-specific may need to be reconsidered, with 16 delta-class GSTs identified from the genome of the two spotted mite, *T. urticae*. The existence of delta-class GSTs in *P. citri* could be related to the close taxonomical relationship between *P. citri* and *T. urticae* in Tetranychidae.

Mu-class GSTs have also been considered vertebrate-specific [33]. In mammals, mu-class GSTs are involved in catalyzing the reaction of glutathione with endo- and xenobiotics, and in a variety of cellular processes like detoxification of endogenous and exogenous compounds in the liver, brain, and testis [34,35] However, the abundance of mu-class GSTs in *S. scabiei* [6], *I. scapularis* [11], *Varroa destructor* and *T. urticae* has been reported [12,36]. Compared with other classes of this gene superfamily, the relative profusion of mu-class GSTs in both *I. scapularis* and *T. urticae* might suggest their more important role in the metabolism of xenobiotics across the whole lifecycle (Table 3). In this study, four complete cDNA sequences encoding mu-class GST genes were obtained. The phylogenetic tree clustered them with mu-class GSTs from *T. urticae* and *I. scapularis* (Figure 1). The deduced amino acid identities between the four *P. citri* mu-class GSTs and the mu-class GSTs in *T. urticae* were 54%–88%, which was higher than in *I. scapularis.* These results were also consistent with their taxonomic status in Tetranychidae.

As in *T. urticae*, only one zeta-class GST was found in *P. citri* (Table 2), and their amino acid sequences shared 75% similarity. In the permethrin-resistant strain of *B. mori*, abundantly distributed zeta-class GSTs possibly contribute to the permethrin resistance of Lepidoptera [37]. However, little information about this class of GSTs in Acari is available.

### 2.3. Developmental Stage-Specific Expression Patterns of GSTs in *P. Citri*

**Figure 2.**
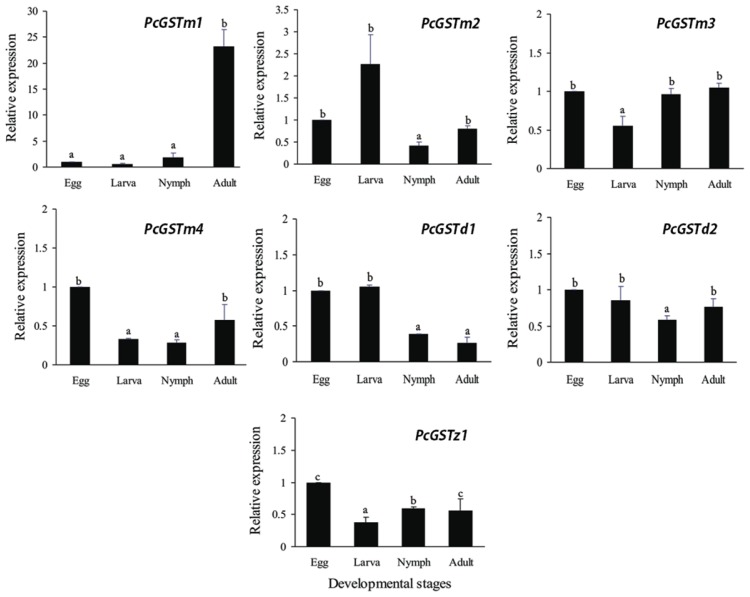
Developmental stage-specific expression patterns of the seven GST genes in *Panonychus citri* were evaluated using RT-qPCR. The following life stages were analyzed: egg, larvae, nymph and adult. The mRNA levels are expressed as mean fold transcription relative to egg. The letters above bars show significant differences among different developmental stages. The *GAPDH* gene was used as a reference. The differences among the four developmental stages were analyzed by one-way analysis of variance (ANOVA), followed by Duncan’s multiple range tests in SPSS 16.0 (SPSS Inc, Chicago, IL, USA).

Developmental stage-specific expression patterns of seven GST genes were examined in eggs, larvae, nymph, and adults using RT-qPCR. Among these seven genes, *PcGSTm1* showed significantly higher expression (23.2-fold) in adult than in other developmental stages (Figure 2). That significant 10 fold greater expression of *PcGSTm1* in adult could be considered as one key factor involved in the rapid development of acaricides resistance. Additionally, a higher relative expression level of GST at the larval stage compared with the adult stage has also been found in *A. gambiae* [38]. However, similar results have not been reported in mites and ticks. Interestingly, four mu-class GST genes (*PcGSTm1*, *PcGSTm2*, *PcGSTm3* and *PcGSTm4*) had various expression patterns in four different developmental stages. However, significantly higher relative expression in adult than in larva was found for all mu-class GSTs, other than *PcGSTm2. PcGSTm3* and *PcGSTm4* showed lower expression in larva than in other stages. The lowest expression for *PcGSTm2* (0.4-fold) was observed in nymph. The two delta-class genes (*PcGSTd1* and *PcGSTd2*) showed similar expression profiles in the four developmental stages and had the lowest relative expression levels in adult and nymph, respectively (Figure 2). In addition, the lowest expression level of *PcGSTz1* (0.37-fold) relative to eggs was observed in the larval stage (Figure 2). In summary, all of the genes were expressed at a higher level in adults than in larva and nymph, except *PcGSTd1*and *PcGSTd2*. These results may be explained by differences in feeding rate and the various environmental challenges caused by mobility differences of *P. citri* during those stages.

### 2.4. Effect of Pyridaben and Fenpropathrin on GST Expression in *P. citri*

Pyridaben, one of the mitochondrial electron transport inhibitor (METI) acaricides, is highly efficient against all life stages of spider mites, and is frequently used in many crops worldwide. METI resistance in *T. urticae* has been reported in several different regions and host plants [39]. The resistance against pyridaben has also been described in several strains of *Panonychus ulmi* [40]. A synergism study with diethylmaleate (DEM) and the measurement of P450 monooxygenase activity suggested the important factor in resistance to METIs is P450s, rather than glutathione *S*-transferases [41]. On the other hand, the susceptibility to pyridaben of *P. citri* has rapidly decreased over a short time in China [19]. Increased GST activity may be one of factors that contribute to the development of pyridaben resistance in *P. citri* [24]. To further reveal the relationship between GSTs and resistance to pyridaben in *P. citri*, we chose pyridaben as one of the two acaricides in this study to induce the expression of GST genes. Meanwhile, as a commonly-used acaricide in many orchards, fenpropathrin has a high resistance risk in *P. citri* [42]. The development of fenpropathrin resistance in *T. urticae* has been suggested to be related to the mutation and deletion of amino acid residues in the sodium channel α-subunit gene (*Tuvssc*) [43]. However, in *P. citri*, elevated activities and/or expression of GST genes associated with resistance to fenpropathrin have not been reported to date.

**Figure 3.**
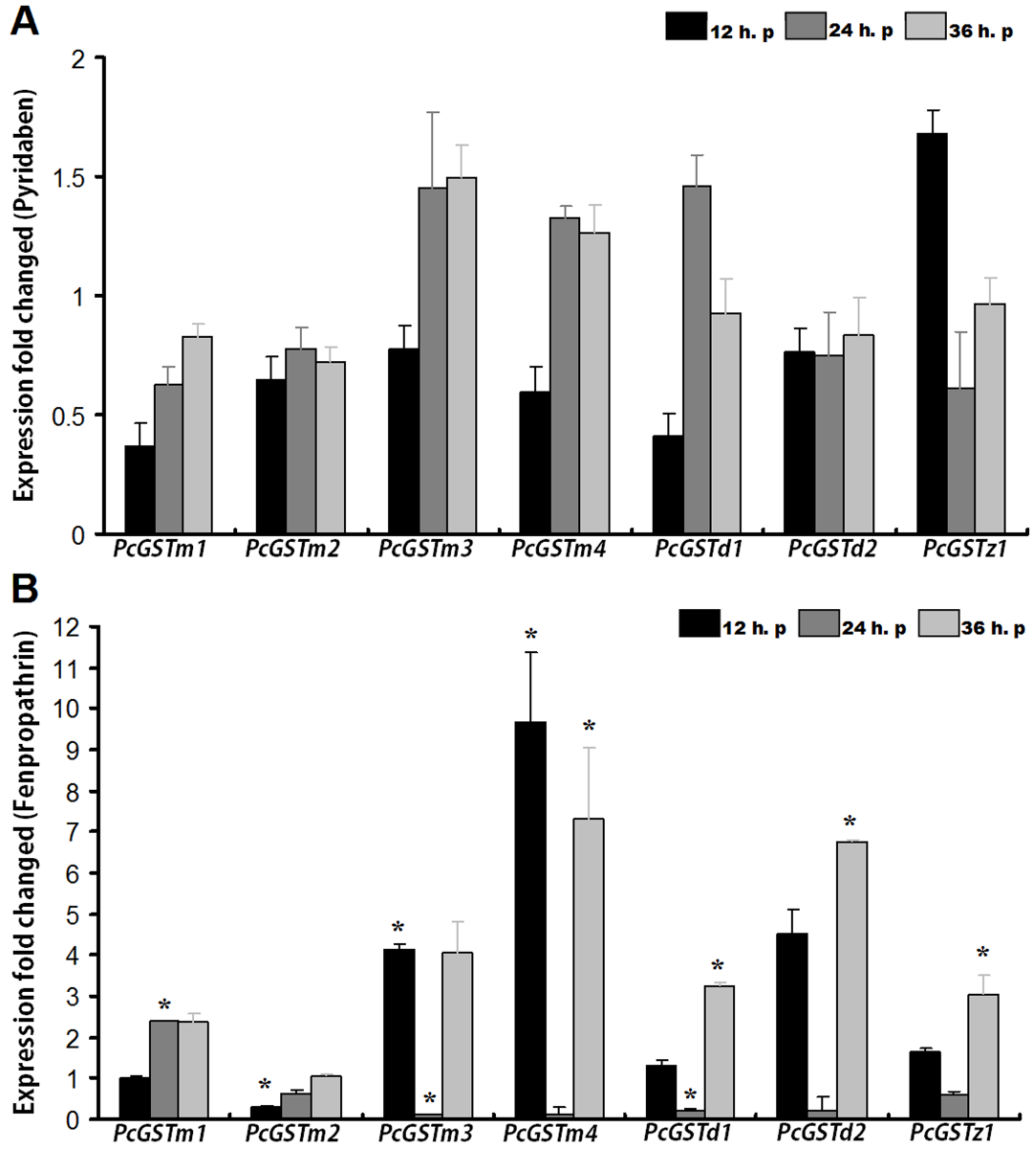
Quantification of relative expression levels of the seven GST genes in female adult *Panonychus citri* exposed to pyridaben (**A**) and fenpropathrin (**B**) at *LC*_10_ for 12, 24 and 36 h post-treatment. The mRNA level in the control and each treatment was normalized using *GAPDH* as a reference gene. The mean expression in each treatment was shown as a fold change compared with the mean expression in the control, which was defined as a basal value of 1. The vertical bars indicate standard errors of the mean (*n* = 3). The asterisks on the bars indicate that means are significantly different among the control and treatments (*p* < 0.05).

In this study, induction results of the two acaricides indicated that none of the seven genes were significantly up-regulated following 12, 24, or 36 h of exposure to pyridaben (Figure 3A). The highest relative expression level was only 1.6-fold after 36 h of exposure to pyridaben at *LC*_10_. In field-collected pyridaben-resistant *P. citri* populations, synergism experiments with DEM, PBO and TPP suggested that GSTs play a major role in the development of pyridaben resistance [44]. In *T. urticae*, however, the inhibition study with DEM suggested that GSTs are not an important factor in resistance to pyridaben [41]. These differences may be explained by inter-specific differences [45]. Although the results in current study are consistent with a previous study in *T. urticae* we could not conclude for certain that GST is not an important factor in pyridaben tolerance, because the response of these seven GST genes to pyridaben exposure cannot completely represent the tendency of the whole supergene family. Therefore, remaining GSTs, especially those belonging to different classes, should be studied to determine whether they can be induced by pyridaben exposure in *P. citri*. Additionally, the concentration of pyridaben and exposure time might also impact the findings and therefore cannot be ruled out.

Interestingly, the relative expression levels of six GST genes (*PcGSTm1*, *PcGSTm3*, *PcGSTm4*, *PcGSTd1*, *PcGSTd2* and *PcGSTz1*) in *P. citri* showed different time-dependent expression profiles when exposed to a sub-lethal concentration of fenpropathrin (Figure 3). Compared with the control, the relative expression levels of mu-class genes *PcGSTm3* and *PcGSTm4* were 4.1-fold and 9.6-fold higher soon after 12 h exposure to fenpropathrin. Another mu-class gene, *PcGSTm1*, also increased (2.4-fold) after 24 h of fenpropathrin exposure. No significantly increased relative expression level was found for *PcGSTm2*, suggesting its minor role in detoxification of fenpropathrin. Two delta-class GST genes, *PcGSTd1* and *PcGSTd2*, were up-regulated 3.2-fold and 6.0-fold after 36 h of fenpropathrin treatment, respectively (Figure 3B). In addition, after 36 h of treatment with fenpropathrin, the relative expression levels of *PcGSTm4* and *PcGSTz1* also showed 7.3-fold and 3.0-fold increases, respectively.

The involvement of GSTs in pyrethroid resistance has been well studied. By binding to the molecule of pyrethroid insecticides in a sequestering mechanism, GSTs offer passive protection against pyrethroid [46]. Elevated GST levels may contribute to pyrethroid-induced lipid peroxidation to protect tissues from oxidative damage, and confer pyrethroid resistance in *N. lugens* [7]. However, little is known about GSTs that are involved in the resistance to pyrethroid acaricides. In the present study, three mu-class and two delta-class GST genes were induced following exposure to fenpropathrin (Figure 3B). This finding suggested that mu-class and delta-class GSTs in *P. citri* could be induced by fenpropathrin, and might play an important role in the development of resistance to fenpropathrin. Similar findings were obtained in permethrin-resistant *S. scabiei* [6], and are also consistent with several studies investigating the role of elevated GST activity in the development of resistance to pyrethroid insecticides in *Aedes aegypti* [47], *Spodoptera littoralis* [48] and *Tribolium castaneum* [49]. In addition that, zeta-class GST *PcGSTz1* was also induced by fenpropathrin, similar to the mu- and delta-class GSTs. A similar result has been reported recently in *B. mori*, wherein GSTs were shown to catalyze the dechlorination of permethrin [36].

However, some of the seven GST genes showed decreased expression: *PcGSTm2* (0.3-fold, 12 h treatment), *PcGSTm3* (0.1-fold, 24 h treatment) and *PcGSTd1* (0.2-fold, 24 h treatment). Among those, *PcGSTm2* expression recovered to generally the same level as the control after 24 and 36 h of fenpropathrin exposure. The non-induction response of *PcGSTm2* to fenpropathrin exposure might suggest the discrepancies among different subclasses of GST genes. Dissimilar responses after various exposure times for these GST genes might be explained by a dynamic balance system to reasonably assign the energy among all the GST genes when challenged by xenobiotics.

The increased expression of GST genes (*PcGSTm1*, *PcGSTm3*, *PcGSTm4*, *PcGSTd1*, *PcGSTd2* and *PcGSTz1*) in *P. citri* might also result in an elevated tolerance to other pesticides and xenobiotics. Although the expression level of the remaining GST gene, *PcGSTm2*, was not significantly higher than the control when exposed to fenpropathrin, we speculate that this enzyme may be involved in resistance to other acaricides that were not tested rather than acaricides tested in the current study. Moreover, GSTs have not been shown to directly metabolize pyrethroid acaricides [2]. Therefore, further functional research of GSTs as detoxification enzymes or protector against resulting oxidative stress caused by xenobiotics is indispensable to elucidate the role of specific GSTs in fenpropathrin resistance development.

## 3. Experimental Section

### 3.1. Mites

The laboratory colony of *P. citri* was collected randomly from the Banco orchard at the Citrus Research Institute, Chinese Academy of Agricultural Sciences, Chongqing, China, in 2012. The collected mites were maintained at 25 ± 1 °C and 60% relative humidity under a 14:10 h light:dark regime. Mites at uniform developmental stages (including egg, larva, nymph and adult) were collected for RNA extraction, according to a previously-described collection method [22]. This population was relatively susceptible to both pyridaben and fenpropathrin based on results of laboratory bioassays together with a field population which has been challenged by acaricides for several years.

### 3.2. Transcriptome Analysis and Identification of *P. Citri* GST cDNAs

The GST genes from *P. citri* were identified by searching the Unigene transcriptome database [50] for keywords (GST, glutathione transferase and glutathione *S*-transferase) or by using the basic alignment search tool (BLAST) algorithm to search for other known arthropod GST genes. Unigene transcripts that were relatively short (<300 bp) were manually removed and the remaining 24 Unigene transcripts were analyzed. To confirm the identity of the transcripts as GST genes, the putative GST cDNAs were subjected to BLASTP analysis to compare the predicted amino acid sequences against the non-redundant database at NCBI [50].

### 3.3. RNA Isolation and First-Strand cDNA Synthesis

Total RNA used for full-length cDNA amplification and analysis of the expression profiles of the GST genes was extracted using an RNeasy Plus Micro Kit (Qiagen GmbH, Hilden, Germany) according to the manufacturer’s instructions. Genomic DNA was removed using a genomic DNA elimination column supplied with the kit. The total RNA from each sample was dissolved in 20 μL of diethylpyrocarbonate-treated water and stored at −80 °C for further analysis. RNA quantity was measured using the absorbance at 260 nm using a Nanovue UV-vis spectrophotometer (GE Healthcare, Fairfield, CT, USA), and the quality was assessed at an absorbance ratio of *OD*_260/280_. The RNA integrity was further confirmed by estimating the 28S/18S rRNA ratio by 2% agarose gel electrophoresis. The RNA for cDNA cloning and RT-qPCR was then reverse transcribed to synthesize first-strand cDNA using a PrimeScript 1st Strand cDNA Synthesis Kit (Takara Biotechnology Co., Dalian, China). The 5′ and 3′ cDNA regions were added using a SMARTer RACE cDNA Amplification Kit (Clontech, Mountain View, CA, USA) following the manufacturer’s instructions.

### 3.4. Cloning of Full-Length GST cDNA

Based on the transcript Unigene sequences obtained from transcriptome searching, gene-specific primers were designed and synthesized (Table S1) for 5′ and 3′ rapid amplification of cDNA ends (RACE) analysis. Two universal primers, UPM (5′-CTAATACGGACTCACTATAGGGCAAG CAGTGGTATCAACGCAGAGT-3′) and NUP (5′-AAGCAGTGGTATCAACGCAGAGT-3′), were also used. A pair of specific primers was designed to amplify the open reading frame for each gene after RACE analysis to confirm the full-length cDNA sequences (Table S2). The PCR conditions were determined empirically for amplification of each GST cDNA. They were 3 min at 95 °C, followed by 34 cycles of 30 s at 95 °C, 30 s at 55–65 °C (depending on gene specific primers) and 60 s at 72 °C, then 10 min at 72 °C. The PCR products were purified from 1% agarose gel by Gel Extraction Mini Kit (Watson Biotechnologies Inc., Shanghai, China) and cloned into a pGEM-T Easy vector (Promega, Fitchburg, MA, USA). Inserts were further sequenced for confirmation (BGI, Beijing, China).

### 3.5. Phylogenetic Analysis

Deduced amino acids of obtained GSTs from *P. citri*, *T. urticae*, *I. scapularis*, and some of other model insects were aligned using ClustalX (v. 2. 0) [51]. The percentages of amino acid identity for each of the GSTs were determined using the MegAlign program (DNASTAR) [52]. The corresponding phylogenetic trees were determined by the neighbor-joining method, with 1000 bootstrap replicates implemented in MEGA 4.0 [53].

### 3.6. Acaricide Exposures

Fenpropathrin and pyridaben were purchased from Sigma-Aldrich (St. Louis., MO, USA) and used to induce GST gene expression according to the leaf disc immersion method [54]. The responses of GST genes to acaricides were investigated by exposing the female adult mites to these two acaricides at LC_10_ (10% of lethal concentration) for 12, 24 and 36 h, respectively. The *LC*_10_ (0.504 μg mL^−1^ for fenpropathrin and 0.250 μg mL^−1^ for pyridaben) for *P. citri* to the two acaricides was determined by leaf-dip bioassays prior to the acaricide treatments. Each leaf disc with 30 mites on the surface was soaked for 5 s in the acaricide solutions. For each treatment, more than 700 surviving mites were collected and three biological replicates were carried out. In addition, a corresponding number of mites were dipped in distilled water for 5 s. All of the collected surviving mites were then rapidly frozen in liquid nitrogen and stored at −80 °C for RNA extraction.

### 3.7. Real-Time Quantitative PCR and Statistical Analysis

All the primers for RT-qPCR were designed in Primer 3.0 [55] according to the full-length cDNAs obtained in this study (Table S3); based on a stability evaluation conducted in our previous study [56]. *GAPDH* was chosen to be the reference gene for RT-qPCR analysis of GST gene expression at four developmental stages and under acaricide exposures. The suitability of the primers for RT-qPCR was evaluated by a pre-experiment using general cDNA template from female adults. The amplification efficiency was determined by constructing a standard curve from a dilution series (1, 1/3, 1/9, 1/27, and 1/81) of the cDNA samples. The RT-qPCR was performed using a Stratagene Mx3000P thermal cycler (Stratagene, La Jolla, CA, USA) with an initial denaturation at 95 °C for 120 s, followed by 40 cycles of 95 °C for 15 s, 60 °C for 30 s and 72 °C for 30 s. The relative expression level of the GST genes was calculated according to the 2^−ΔΔ^*^Ct^* method [57]. The differences among the four developmental stages were analyzed by one-way analysis of variance. The fold change of GST gene expression level was analyzed using SPSS (v.16.0, SPSS Inc, Chicago, IL, USA), and the significance was determined by independent-sample *t*-test with a *p*-value < 0.05.

## 4. Conclusions

In this study, the full-length cDNA sequences of seven GST genes were identified from *P. citri*. Based on sequence analysis, these genes were placed into three different cytosolic classes, including four in mu, two in delta and one in zeta. In contrast to the dataset of the *T. urticae* genome, more GST genes in *P. citri* are expected to be identified in subsequent studies. Both increases and decreases in expression levels of the GST genes were observed when exposed to fenpropathrin at a sub-lethal concentration, whereas none of the seven GST was induced by pyridaben. These results may be caused by different mechanisms in the resistance development of *P. citri* to these two different types of acaricides/insecticides. Our work provides the first insight into the molecular characteristics and transcription profiles of *P. citri* GSTs when exposed to acaricides/insecticides.

## Supplementary Information



## Figures and Tables

**Table 1.** Overview of molecular properties of seven *P. citri* GSTs.

Gene name	GenBank accession number	Length of cDNA (bp)	ORF (bp)	Number of deduced amino acid residues	Molecular weight (kDa)	*pI*
*PcGSTm1*	JQ069034	1289	675	224	26.5	7.65
*PcGSTm2*	JQ069035	1110	676	224	26.2	5.99
*PcGSTm3*	JX846609	957	669	222	25.9	5.24
*PcGSTm4*	JX846610	915	678	225	26.6	5.18
*PcGSTd1*	JQ069033	905	654	217	23.9	6.52
*PcGSTd2*	JQ069037	894	654	217	24.3	5.45
*PcGSTz1*	JQ069036	822	657	218	24.7	6.91

**Table 2.** Percentage identities of amino acid residues among the seven *P. citri* GST genes.

	*PcGSTm1*	*PcGSTm2*	*PcGSTm3*	*PcGSTm4*	*PcGSTd1*	*PcGSTd2*	*PcGSTz1*
***PcGSTm1***	-	33.04	55.36	55.11	15.35	17.90	11.40
***PcGSTm2***	-	-	37.89	35.24	19.57	18.10	17.30
***PcGSTm3***	-	-	-	60.00	14.67	16.81	17.54
***PcGSTm4***	-	-	-	-	13.10	13.97	14.89
***PcGSTd1***	-	-	-	-	-	53.21	24.45
***PcGSTd2***	-	-	-	-	-	-	24.56
***PcGSTz1***	-	-	-	-	-	-	-

**Table 3.** Comparison of the number of the GST genes from various insects and Acari.

Cytosolic GSTs	Insecta				Acari		

	*D. melanogaster*	*A. gambiae*	*T. castaneum*	*B. mori*	*T. urticae*	*I. scapularis*	*P. citri*
Delta	11	12	3	4	16	7	2
Epsilon	14	8	19	8	-	5	-
Mu	-	-	-	-	12	14	4
Omega	5	1	4	4	2	3	-
Sigma	1	1	7	2	-	-	-
Theta	4	2	1	1	-	-	-
Zeta	2	1	1	2	1	3	1
Unknown	-	3	-	2	-	-	-
Total	37	28	35	23	31	32	7
