# Supplementary material for: Identification and Characterization of Seven Glutathione S-Transferase Genes from Citrus Red Mite, Panonychus citri (McGregor)
Source: Int J Mol Sci. 2013 Dec 13;14(12):24255–70. doi: 10.3390/ijms141224255 (PMC3876109; doi:10.3390/ijms141224255)
Supplement: Supplementary file 1 [file ijms-14-24255-s001.pdf]

Supplementary Information

Table S1. Primers for RACE of seven GST genes of *Panonychus citri*.

Gene name	GenBank accession number	Sequences of primers (5'-3')
PcGSTm1	JQ069034	5'RACE: CGGTATGATTGCTTGAGTTG
		3'RACE: TCCTTCGTTACATTGGTCG
PcGSTm2	JQ069035	5'RACE: AGAAGAAGACGAATGGGTT
		3'RACE: CCTAATCTTCCCTATTTCATCG
PcGSTm3	JX846609	5'RACE: TGGAGCACCACCAAGTT
		3'RACE: ACTCAGACTTTGGCGATTC
PcGSTm4	JX846610	5'RACE: TAACGGAGAATAGCCAAAG
		3'RACE: AACATACATCGCAAGTTCTGG
PcGSTd1	JQ069033	5'RACE: TGGTCTTCTCCAATGAACTGTC
		3'RACE: GATTTGGACAGTTCATTGGAGA
PcGSTd2	JQ069037	5'RACE: TCATCAACCAAGGTAGGG
		3'RACE: GGACAATGGGTCACTTTACG
PcGSTz1	JQ069036	5'RACE: TGATGAATGAAACCAAACG
		3'RACE: ATGCTTATCGTTATGGTGTCG

Table S2. Primers for full-length confirmation of seven GST genes from *Panonychus citri*.

GenBank	Gene	Sequences of primers (5'-3')
JQ069034	*PcGSTm1*	M1FS: ATGGCTCCAACTATCGGTTATTGGC
		M1FA: TCATGTTGATTGATTGTTTCCCCATC
JQ069035	*PcGSTm2*	M2FS: ATGGCTCCTGTTATTGGTTATTG
		M2FA: TTACTCCTCCGGAGGATTGTGTCG
JX846609	*PcGSTm3*	M3FS: ATGGCCCCGATTCTTGGCTACT
		M3FA: CTAAGTTTGGAAGCGGAAATTAG
JX846610	*PcGSTm4*	M4FS: ATGACACCAATTATTGGTTATTG
		M4FA: TTAGTTAGACGGAGGATTGTGGC
JQ069033	*PcGSTd1*	D1FS: ATGTCCATCCAATTGTTTC
		D1FA: TTATTTTGAAGCTTTGGATT
JQ069037	*PcGSTd2*	D2FS: ATGGTCTTGGAATTGTATCAAT
		D2FA: TTACTGAGCAAGTTTTGATTT
JQ069036	*PcGSTz1*	Z1FS: ATGTCCAAGGTGAAATTATTTTC
		Z1FA: CTATTGTTTTGGTGCATCGGGTTG

Table S3. Primers for RT-qPCR of GST genes of *Panonychus citri*.

GenBank	Gene	Sequences of primers (5'-3')
JQ069034	*PcGSTm1*	M1qPCRs: TGGACCCAAATCTGAAGAGG
		M1qPCRa: AAGGGCAGTTGATGTTTGCT
JQ069035	*PcGSTm2*	M2qPCRs: GGCGAGAATAAGTTCGTTGC
		M2qPCRa: TGCTCGATCACCATTGAGAG
JX846609	*PcGSTm3*	M3qPCRs: ACTTGGTGGTGCTCCAGATT
		M3qPCRa: CCTCTTCTCCTTTGGGTCCT
JX846610	*PcGSTm4*	M4qPCRs: ACATCGCAAGTTCTGGGTTC
		M4qPCRa: GATCCGATGATTGGAAATCG
JQ069033	*PcGSTd1*	D1qPCRs: GAGTCGCTCGAGTTTTGGTC
		D1qPCRa: GAATTACCTGGGGCGAGTTT
JQ069037	*PcGSTd2*	D2qPCRs: GGAGCTAAACCCGATCCTTC
		D2qPCRa: CTGGATCGGTGACCAGTTCT
JQ069036	*PcGSTz1*	Z1qPCRs: CCGTTTGGTTTCATTCCATC
		Z1qPCRa: TGGTCTTTATTTCCGCCAAG
HM582445	*GAPDH*	GqPCRs: CTTTGGCCAAGGTCATCAAT
		GqPCRa: CGGTAGCGGCAGGTATAATG

© 2013 by the authors; licensee MDPI, Basel, Switzerland. This article is an open access article distributed under the terms and conditions of the Creative Commons Attribution license (http://creativecommons.org/licenses/by/3.0/).
